# Toward harmonized phenotyping of human myeloid-derived suppressor cells by flow cytometry: results from an interim study

**DOI:** 10.1007/s00262-015-1782-5

**Published:** 2016-01-04

**Authors:** Susanna Mandruzzato, Sven Brandau, Cedrik M. Britten, Vincenzo Bronte, Vera Damuzzo, Cécile Gouttefangeas, Dominik Maurer, Christian Ottensmeier, Sjoerd H. van der Burg, Marij J. P. Welters, Steffen Walter

**Affiliations:** 1 Section of Oncology and Immunology, Department of Surgery, Oncology and Gastroenterology, University of Padova, Via Gattamelata, 64, 35128 Padua, Italy; 2 Veneto Institute of Oncology IOV - IRCCS, Padua, Italy; 3 Department of Otorhinolaryngology, University Hospital Essen, Essen, Germany; 4 TRON Translationale Onkologie an der Universitätsmedizin der Johannes Gutenberg-Universität Mainz GmbH, Mainz, Germany; 5 Present Address: Cell Therapy Group, Immuno-Oncology and Combinations, GlaxoSmithKline, Stevenage, UK; 6 Section of Immunology, Department of Pathology and Diagnostics, Verona University Hospital, Verona, Italy; 7 Department of Immunology, Institute for Cell Biology, University of Tübingen, Tübingen, Germany; 8 Immatics Biotechnologies GmbH, Tübingen, Germany; 9 Cancer Sciences Unit, Faculty of Medicine, Experimental Cancer Medicine Centre, Southampton General Hospital, University of Southampton, Tremona Road, Southampton, UK; 10 Department of Clinical Oncology, Leiden University Medical Center, Leiden, The Netherlands

**Keywords:** Myeloid-derived suppressor cells, Phenotyping, Proficiency panel, Flow cytometry

## Abstract

There is an increasing interest for monitoring circulating myeloid-derived suppressor cells (MDSCs) in cancer patients, but there are also divergences in their phenotypic definition. To overcome this obstacle, the Cancer Immunoguiding Program under the umbrella of the Association of Cancer Immunotherapy is coordinating a proficiency panel program that aims at harmonizing MDSC phenotyping. After a consultation period, a two-stage approach was designed to harmonize MDSC phenotype. In the first step, an international consortium of 23 laboratories immunophenotyped 10 putative MDSC subsets on pretested, peripheral blood mononuclear cells of healthy donors to assess the level of concordance and define robust marker combinations for the identification of circulating MDSCs. At this stage, no mandatory requirements to standardize reagents or protocols were introduced. Data analysis revealed a small intra-laboratory, but very high inter-laboratory variance for all MDSC subsets, especially for the granulocytic subsets. In particular, the use of a dead-cell marker altered significantly the reported percentage of granulocytic MDSCs, confirming that these cells are especially sensitive to cryopreservation and/or thawing. Importantly, the gating strategy was heterogeneous and associated with high inter-center variance. Overall, our results document the high variability in MDSC phenotyping in the multicenter setting if no harmonization/standardization measures are applied. Although the observed variability depended on a number of identified parameters, the main parameter associated with variation was the gating strategy. Based on these findings, we propose further efforts to harmonize marker combinations and gating parameters to identify strategies for a robust enumeration of MDSC subsets.

**Electronic supplementary material:**

The online version of this article (doi:10.1007/s00262-015-1782-5) contains supplementary material, which is available to authorized users.

## Introduction

Myeloid-derived suppressor cells (MDSCs) are a heterogeneous group of myeloid cells at different stages of differentiation that are often expanded in cancer patients and capable to suppress immune responses [1]. In recent years, recognition of the clinical relevance of MDSCs has steadily increased. MDSC levels correlate negatively with prognosis and overall survival in cancer patients, and the accumulation of this population promotes tumor progression and is linked to tumor stage and worse prognosis of cancer patients [2–5].

MDSCs were originally described in mice, on the basis of the co-expression of CD11b and Gr-1. However, subsequent studies elucidated that CD11b^+^/Gr-1^+^ cells are not a homogeneous cell population, but rather a heterogeneous group of myeloid cells, endowed with different suppressive ability. At present, it is well known that there are at least two main subsets of MDSCs present in tumor-bearing mice, the monocytic (M-MDSC) and the granulocytic (G-MDSC) ones and that these cell populations can be properly identified by using a set of accessory markers. In particular, the differential expression of Ly6C and Ly6G, the two isoforms of Gr-1, allows the identification of G-MDSC as CD11b^+^/Gr-1^high^/Ly6C^−^/Ly6G^high^ and M-MDSC as CD11b^+^/Gr-1^int^/Ly6C^high^/Ly6G^−^ [6].

The phenotyping of human MDSCs is complicated by the fact that Gr-1 is not expressed on human leukocytes. Thus, multiple human MDSC subsets with different phenotypes have been documented in several types of tumors in the last 20 years (reviewed in [7]). All reported phenotypes are defined on the basis of a combination of myeloid markers and suffer from the lack of a specific marker. The initial assumption that MDSCs are solely constituted of immature myeloid cells [8–10] is now challenged by reports describing MDSCs with morphology and phenotype consistent with those of cells with more differentiated features. At present, it appears that all MDSC phenotypes can be allocated to one of the at least three main subsets, of which each contains more than one cell population. M-MDSCs share the morphology of monocytes and are characterized by the expression of CD14 and lack of CD15 [11, 12]; G-MDSCs contain a cell population resembling granulocytes and are phenotypically characterized by CD15 and CD66b expression and the absence of CD14 [12–16]; immature MDSCs (i-MDSC) are cells with immaturity characteristics, defined essentially as lineage negative cells [3, 17].

Although there is an increasing relevance of MDSCs for tumor immunology, there is lack of a uniformly used and broadly accepted classification of human MDSCs (in terms of both subsets types and identification markers). This motivated the design of a proficiency panel by the CIMT (Association for Cancer Immunotherapy) Immunoguiding Program (CIP) (http://www.cimt.eu/workgroups/cip/) to assess the heterogeneity in current human MDSC phenotyping approaches and harmonize them across different laboratories. The first step of the proficiency panel, which is the focus of this communication, did not include a functional assay, since the suppressive activity of most of these subsets was already reported [7]; hence, this study was mainly designed to assess intra-laboratory and inter-laboratory variance and to define a robust marker combination for the phenotypical identification of circulating MDSCs. The parameters associated with high variance identified from this first phase will be harmonized in the second step with the aim to decrease the variability of MDSC quantification.

## Materials and methods

### Healthy donors

After given written informed consent, leukapheresis was performed for 23 healthy individuals. Peripheral blood mononuclear cells (PBMCs) were centrally prepared by Ficoll gradient and cryopreserved as described previously [4]. The healthy donors were screened by flow cytometry for the presence of the 10 putative MDSC subsets by the organizing committee, and three representative donors with the most significant expansion of these subsets were selected for the first phase of the proficiency panel.

### Proficiency panel

**Fig. 1:**
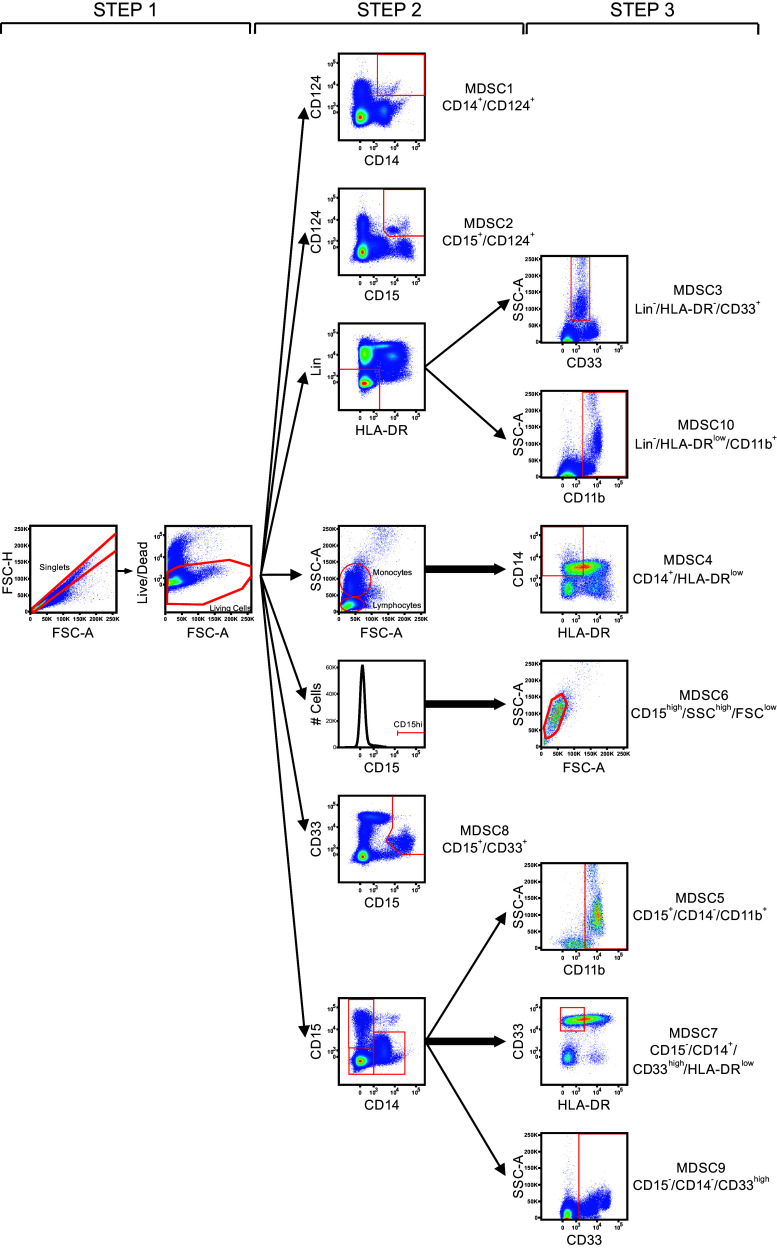
Exemplary gating strategy included in the panel’s guideline. The figure shows the gating strategy proposed in the guidelines and made of three sequential steps, allowing the identification of the 10 putative MDSC subsets. *Step 1* consists in doublets exclusion followed by dead-cell exclusion. *Step 2* allows the identification of three MDSC subsets (MDSC1-2-8), and from a consecutive gate, the other seven defined MDSC populations are identified in *step 3*

Two vials of each donor were shipped to the participant laboratories in dry ice and stored in liquid nitrogen upon arrival. The participating laboratories were asked to perform the immunophenotyping of 10 putative subsets of MDSCs on the cryopreserved samples from the three selected healthy donors (HBC-480, HBC-514, L29-3). Experiment guidelines to indicate the mandatory parameters to be fulfilled and with exemplary staining cocktails, protocol and gating strategy (Fig. 1) were provided to the participants. The proposed staining cocktails contain 7 markers commonly used for MDSC recognition (as deduced from two webinars held on MDSCs by CIP) plus a dead-cell marker (DCM): HLA-DR, CD14, CD15, CD11b, CD33, lineage cocktail (defined as CD3/14/19/56) and CD124. This marker combination allows the identification of 10 putative MDSC subsets: MDSC1 CD14^+^/CD124^+^ [12]), MDSC2 CD15^+^/CD124^+^ [12], MDSC3 Lin^−^/HLA-DR^−^/CD33^+^ [10], MDSC4 CD14^+^/HLA-DR^low/−^ [11], MDSC5 CD15^+^/CD14^−^/CD11b^+^ [15], MDSC6 CD15^+^/FSC^low^/SSC^high^ [13], MDSC7 CD15^−^/CD14^+^/CD33^high^/HLA-DR^low^ [18], MDSC8 CD15^+^/CD33^high^, MDSC9 CD14^−^/CD15^−^/CD33^high^ and MDSC10 Lin^−^/HLA-DR^low^/CD11b^+^ [19]. Participants were asked to perform the staining twice, in two separate days, in order to calculate intra-laboratory variance. For each donor in each of the two experimental runs, participants were asked to determine cell viability after thawing, test and report the number of total cells, singlets, monocytes and lymphocytes plus 10 putative MDSC phenotypes using one 8-color panel and/or three 4-color antibody flow cytometry panels.

In addition, guidelines indicated to use at least one million PBMCs per test, not to perform a resting period after thawing the cells and not to fix the stained cells.

Participants were asked to analyze the flow data using their own gating strategy and to report it in single layouts; moreover, they were asked to determine the absolute number of the requested cell populations with or without the presence of a DCM.

### Central data analysis

Results from the 23 participating laboratories were centrally collected and analyzed. Absolute numbers of the 10 putative MDSC subsets were normalized to the count of lymphocytes + monocytes. Variance was calculated as follows: %CV = (standard deviation/mean)*100. Comparison between %CV or normalized frequencies of myeloid subsets identified by different groups was performed using the Wilcoxon signed-rank test. Significance level was set for *P* < 0.05.

## Results

To start this project, we conducted a survey by sending out a questionnaire to groups that the organizers identified as having published at least one paper on human MDSCs. We explored their interest in participating to a harmonization process for the phenotyping of human MDSCs. Results of 89 analyzed questionnaires were discussed at the CIMT Annual Meeting 2012 and in two webinars hold in September 2012 with more than 50 participants. Most research groups expressed strong interest to join this activity and, in particular, to reach an agreement for the markers required to phenotype MDSCs. From the initial survey, it appeared that overall 32 markers were used to define MDSCs, but only a core set of 7 markers was commonly used by all groups.

On the basis of these results, a consensus could be reached among the webinar participants to define the following 7 markers describing 10 different putative subsets of MDSCs for further testing: MDSC1 CD14^+^/CD124^+^ [12], MDSC2 CD15^+^/CD124^+^ [12], MDSC3 Lin^−^/HLA-DR^−^/CD33^+^ [10, 20], MDSC4 CD14^+^/HLA-DR^low/−^ [11], MDSC5 CD15^+^/CD14^−^/CD11b^+^ [15], MDSC6 CD15^+^/FSC^low^/SSC^high^ [13], MDSC7 CD15^−^/CD14^+^/CD33^high^/HLA-DR^low/−^ [18], MDSC8 CD15^+^/CD33^high^, MDSC9 CD14^−^/CD15^−^/CD33^high^ and MDSC10 Lin^−^/HLA-DR^low/−^/CD11b^+^ [19]. Lineage (Lin) was defined by the cocktail of anti-CD3/14/19/56 antibodies.

For ethical and practical reasons, it was not feasible to obtain leukaphereses from cancer patients, and therefore, PBMCs of healthy donors were used for this harmonization panel. Participants were asked to phenotype the samples for the 10 predefined putative MDSC subsets. A total of 23 laboratories (15 European and 8 from the USA) participated to the proficiency panel and reported their results to the organizing committee.

Participants were asked to stain the PBMC with antibodies against HLA-DR, CD14, CD15, CD11b, CD33, lineage cocktail (defined as specified above) and CD124 plus a dead-cell marker (DCM). The participating laboratories chose gating strategy, antibody clones and fluorochromes, protocol for staining, negative controls and the inclusion of additional markers. An exemplary, but optional, gating strategy was provided in the guidelines (Fig. 1). We mandated that centers would use either one 8-color antibody panel or three 4-color antibody panels to combine the 8 proposed markers and to perform two experimental runs for each donor on different days. As readout, participants reported the number of total events, lymphocytes, monocytes and MDSC subsets in either the presence or absence of DCM.

For each MDSC subset and each donor, we calculated the intra-assay and inter-assay variance (%CV) of each reported MDSC subset frequency.

**Fig. 2:**
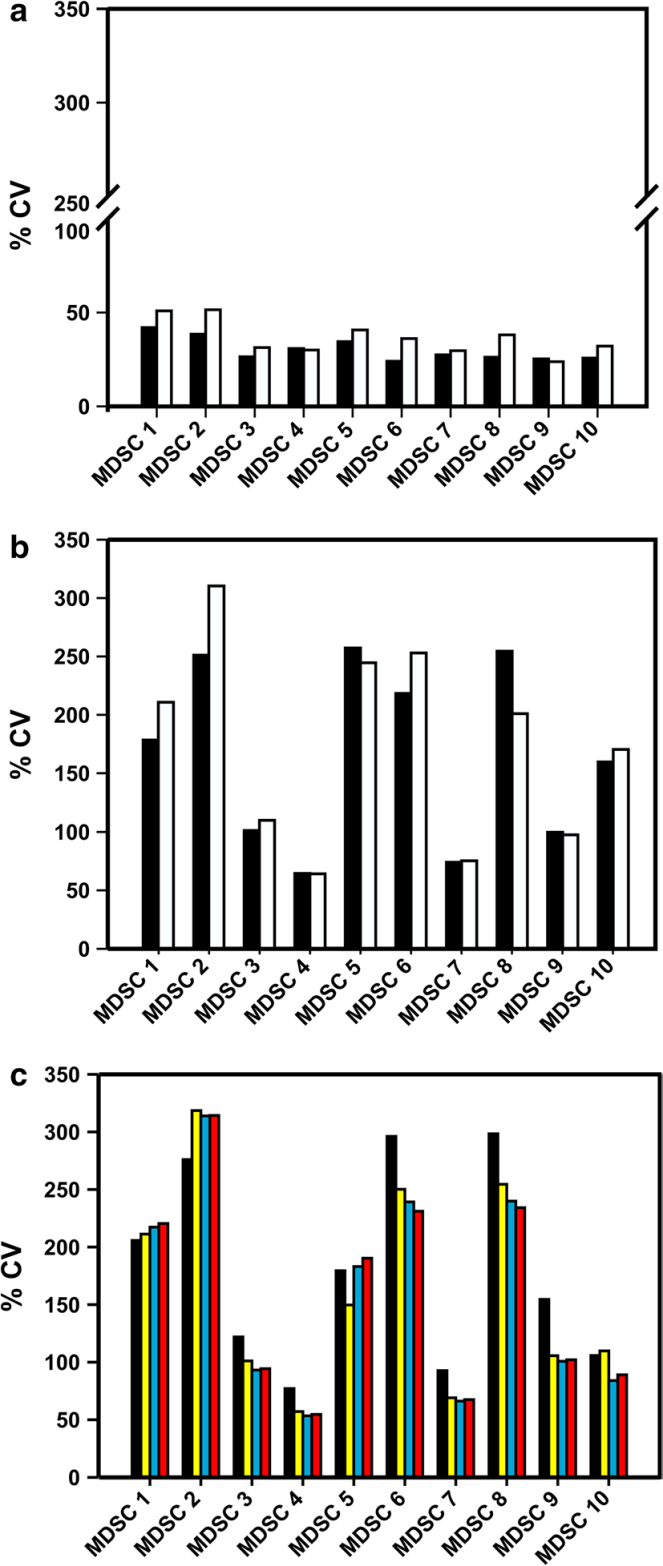
Analysis of the impact of a dead-cell marker on MDSC quantification. *Black bars* indicate average %CV from analyses performed without exclusion of dead cells, while* white bars* refer to analyses considering only live cells (*n* = 18 evaluable laboratories reporting data for the 8-color panel). Intra-laboratory variance is shown in panel **a,** while panel **b** reports inter-laboratory variance. **c** Average %CV (*n* = 14 evaluable laboratories reporting data for the 8-color panel) of the frequencies of the ten MDSC subsets without normalization (*black bar*) and normalized on the number of singlets (*yellow bar*), lymphocytes (*blue bar*) and lymphocytes + monocytes (*red bar*)

**Fig. 3:**
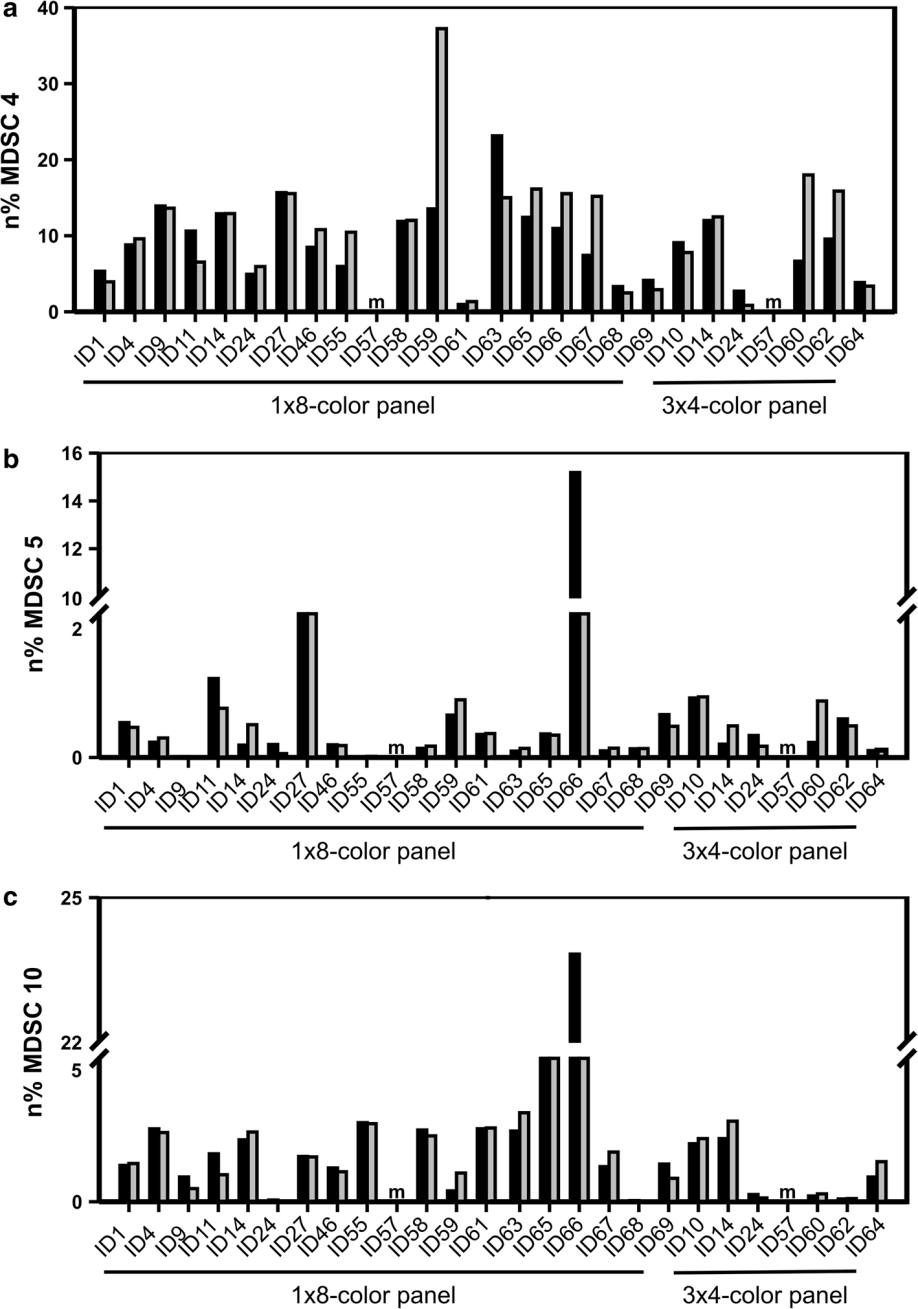
Values of a representative subsets of monocytic (MDSC4 – panel **a**), granulocytic (MDSC5 – panel **b)** and immature (MDSC10 – panel **c**) MDSCs are shown as examples. Frequencies were normalized on the count of lymphocytes + monocytes for each laboratory performing either one 8-color panel or three 4-color panels for MDSC identification; the two paired histograms refer to the two independent experimental runs performed by each laboratory (*black bars* = first run, *gray bars* = second run). Missing values, not reported by single laboratories, were labeled with “*m*”

Intra-laboratory variance was as expected for multicolor phenotyping assays within a lower range (range 20–45 %, Fig. 2a). However, data analysis revealed large inter-laboratory variance among the 10 MDSC subsets analyzed, ranging from 50 to 300 %, with the highest variance among the granulocytic subsets compared to the immature and monocytic subsets (Fig. 2b). When analyzing the influence of data normalization, we found that normalizing MDSC frequencies (*n*% MDSC) to either lymphocytes or lymphocytes + monocytes generally reduced variance (Fig. 2c). Exemplary data of individual laboratories for three subsets are shown in Fig. 3 (panels a, b and c), and the descriptive statistics of the results is reported in Supplementary Table 1.

**Fig. 4:**
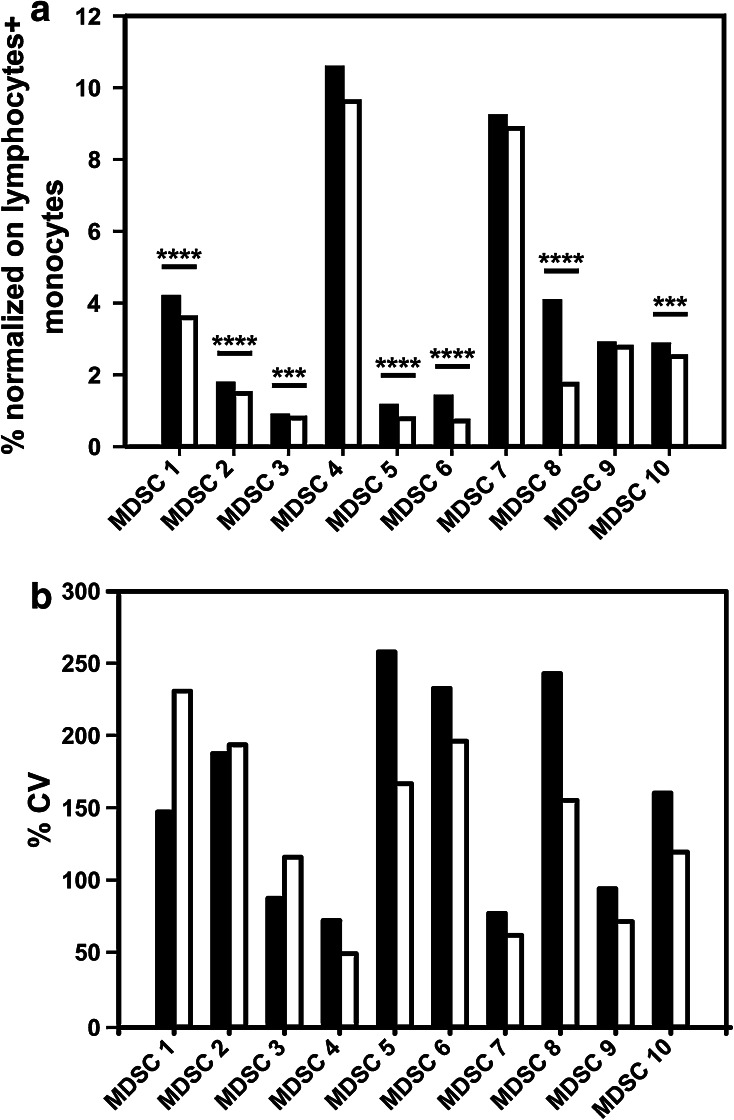
**a** Average frequencies (*n* = 18 evaluable laboratories reporting data for the 8-color panel) of MDSC subsets normalized on lymphocytes + monocytes (* n*%) identified either excluding or not the dead cells. Wilcoxon matched-pair test (***P < 0.001, ****P < 0.0001). **b** Average %CV (*n* = 18 evaluable laboratories reporting data for the 8-color panel) of the frequencies of MDSC subsets identified either using a homogenous gating strategy (*white bar*) or not (*black bars*)

In view of the large spread of the data, we set out to identify the potential sources of variability in the phenotyping of the 10 MDSC subsets. Accordingly, we analyzed the* n*% MDSC and the %CV obtained from the analysis performed with or without DCM. Results indicate that the use of DCM decreases the * n*% MDSC of G-MDSC (MDSC2, 5, 6 and 8), M-MDSC MDSC1 and i-MDSC (MDSC3 and 10) but not of monocytic subsets MDSC4 and 7 (Fig. 4a). The explanation for these results probably lies on the fragile nature of granulocytic cells, which are more prone to cell death during the cryopreservation and/or thawing procedure than either monocytic subsets or lymphocytes [6, 21]. The use of a dead-cell marker did not significantly affect the variance of results obtained from two tests performed by the same laboratory (intra-laboratory; Fig. 2a), while it increased the inter-laboratory variance of results (Fig. 2b; *P* < 0.0001, Wilcoxon matched-pair test).

Other parameters, such as antibody clones and the use of 8-color versus 4-color panels, were difficult to analyze, because the large number of subgroups identified for each variable led to numbers too small for statistical approaches. However, results suggest that the choice of negative controls, CD124 clone and composition of the lineage cocktail were associated with high inter-laboratory variation (data not shown).

It is well known that one of the sources of inter-laboratory variability arises from manual gating analysis of flow cytometry data [22–24], and, indeed, we observed a large variability in the gating strategy developed by the participating groups. For example, some participants put the gate for CD124^+^ cells only on highly fluorescent cells, while others gated positive cells including also dimly fluorescent cells; as a consequence, total %CV for MDSC1 is twofold higher than the %CV calculated within each of the two groups that use a similar gating strategy to identify CD124^+^ cells (data not shown). Also the gating of CD33 positive cells was differently performed including CD33 low and/or high cells, thereby increasing the variance. To dissect the influence of gating analysis on the result variance, we grouped the laboratories that followed the gating example provided in our guidelines (Fig. 1) for the identification of the 10 MDSC subsets. We observed that for those participant results following a homogeneous gating approach, the %CV was reduced (*P* = 0.0012, Wilcoxon matched-pair test) for most MDSC subsets, especially for G-MDSCs (Fig. 4b).

## Discussion

Circulating levels of MDSCs have been correlated with tumor burden and overall survival in different types of cancer, and some studies demonstrated their prognostic role for the outcomes of different chemotherapeutic regimens (reviewed in [7]). Therefore, it is a promising biomarker now also for response to immunotherapy. However, the validation of the predictive significance of MDSCs in multicenter studies is complicated by the phenotypic complexity of human MDSCs, thereby creating a challenge in finding a consensus on the minimal requirements for MDSC monitoring. To address this challenge, we organized the first proficiency panel to harmonize human MDSC phenotyping.

The task was challenging because of the large number of participants, the complexity of the staining panel and the number of subsets to be identified. Under these circumstances, it is not surprising that the quantification of the 10 requested MDSC subsets was characterized by a high inter-laboratory variance, increasing from monocytic to immature and granulocytic subsets. The number and the international origin of the participating laboratories indicate that the high inter-laboratory variance observed in the panel could be similar as in independent studies on human MDSC published. In contrast, the intra-laboratory variance was in general not high, indicating that the participating laboratories were experienced in the field of multicolor flow cytometry.

The choice of reagents and the gating strategy were critical parameters that impacted on the quantification of MDSC. The participating laboratories had been allowed to decide on staining reagents. As a result, a considerable number of different antibodies were used to identify MDSC subsets using an 8-color staining panel. We observed that the clone of anti-CD124 antibody and the composition of the lineage cocktail significantly influenced the quantification of CD124^+^ MDSCs and immature MDSCs, respectively. The issue of the heterogeneity of reagents was also present in previous proficiency panels, in which a partial standardization of reagents had been suggested [25], especially for culturing of PBMCs intended to be used for functional *T* cell assays [23, 26, 27]. The MDSC proficiency panel introduced the use of a DCM, and, as expected, we observed that the percentages of several MDSC subsets, and in particular of granulocytic ones, were significantly reduced by dead-cell exclusion. Hence, these results raise the question whether dead MDSCs should also be evaluated in frozen samples to account for their presence in fresh samples, or in contrast, whether inclusion of dead cells would alter MDSC quantification due to unspecific binding of antibodies to other cell types.

The gating strategies used for identification of MDSC subsets had a significant influence on the variance of results. Inter-laboratory performance improved significantly when analysis was limited to only those laboratories, which applied a homogenous gating strategy similar to that proposed in the panel’s guidelines. This was not unexpected since most proficiency panels based on flow cytometry similarly recommend harmonization of the gating strategies across laboratories. In a multimer-based proficiency panel, results were audited in order to exclude wet laboratories which regularly used a wrong gating procedure [24, 25]. However, since identification of MDSC subsets derives from a complex combination of markers, we believe that a more adequate approach would be to train the participating laboratories to perform a properly gating pipeline through an in silico panel, as done in ICS proficiency panels [23, 28]. Hence, we are currently setting up an in silico panel to measure the influence of manual gating analysis on the variance of the results. In this study, participants will be asked to re-analyze their own data, as well as those of a supplementary reference dataset, following guided instructions for sequential gating. The output will guide us to design the second step of the proficiency panel in which harmonization should be achieved following a wider set of mandatory requests, dealing not only with gating procedure but also with other parameters such as the use of a DCM, a unique lineage cocktail, and fluorescence-minus-one controls for dimly fluorescent antigens.

## Conclusion

Overall, this interim report emphasizes the lack of a robust definition for human MDSC subsets and highlights the importance of finding an agreement on the harmonized staining and analysis strategy and of translating our findings in general guidelines for immune phenotyping of these cells. We demonstrated that it is feasible to conduct a proficiency panel including a large number of participants and identifying a high number of myeloid subsets using 8-color flow cytometry. In this first step, we provided individual feedback of performance for each laboratory and we identified a number of parameters that must be harmonized in the second step, hence establishing the foundation for the development of a robust assay for MDSC phenotyping.

### Electronic supplementary material

Below is the link to the electronic supplementary material.

Supplementary material 1 (PDF 261 kb)

## Appendix: panel participants

K. Bruderek, S. Brandau, University Hospital Essen, Otorhinolaryngology, Essen, Germany

I. Shevchenko, V. Umansky, Skin Cancer Unit, German Cancer Research Center, Heidelberg and Department of Dermatology, Venereology and Allergology, University Medical Center Mannheim, Ruprecht-Karl University of Heidelberg, Mannheim, Germany

C. Diaz, University of Miami, Miami, FL, USA

D. Riemann, B. Seliger, Martin Luther University Halle, Halle, Germany

A. Martens, H. Zelba, G. Pawelec, Center for Medical Research, University of Tübingen, Tübingen, Germany

M. Tazzari, A. Cova, C. Castelli and L. Rivoltini,

Unit of Immunotherapy of Human Tumors, Fondazione IRCCS Istituto Nazionale dei Tumori, Milan, Italy

R. Rae, S. Attig, Medizinische Klinik III, Universitätsmedizin Mainz, Mainz, Germany

D. Wycze, P. Rodri, A. Ochoa, Louisiana State University, New Orleans, LA, USA

A.R. Coleman, A. Cazaly, D. Joseph-Pietras, University of Southampton, Southampton, UK

J. Vella, P. Serafini, Dept. of Microbiology and Immunology, University of Miami, Miami, FL, USA

Y.P. De Coana, R. Kiessling, M. Nystrom, Karolinska Institute, Solna, Sweden

P. Rayman, Y. Yang, J. Finke, Cleveland Clinic, Cleveland, OH, USA

G. Winkels, K. Bublitz, Miltenyi Biotec, Bergisch Gladbach, Germany

S.R. Hadrup, M. Idorn, University Hospital Herlev, Herlev, Denmark

C. Alanio, M. Albert, L. Rogge, M. Hasan, Center for Human Immunology, Institut Pasteur, Paris, France

J. Hermes, I. Poschke, DKFZ, Heidelberg, Germany

T. Condamine, D. Gabrilovich, The Wistar Institute, Philadelphia, PA, USA

M.J.P. Welters, V.V. Visconti, J.J. Van Ham, S.H. van der Burg, Leiden University, Leiden, The Netherlands

C. Ma, F. Korangy, T. Greten, NCI, Gastrointestinal Malignancy Section, Bethesda, MD, USA

J. Staats, J. Enzor, Duke CFAR Flow Core Facility, Durham, NC, USA

L.H. Butterfield, M.J. Buffo, University of Pittsburgh Cancer Institute Immunologic Monitoring Lab, Pittsburgh, PA, USA

V. Damuzzo, S. Mandruzzato, University of Padova, Padova, Italy

D. Maurer, S. Walter, Immatics Biotechnologies GmbH, Tübingen, Germany

## Abbreviations

CIMT: Association for Cancer Immunotherapy
CIP: CIMT Immunoguiding Program
DCM: Dead-cell marker
G-MDSC: Granulocytic MDSC
i-MDSC: Immature MDSC
M-MDSC: Monocytic MDSC
*n*% MDSC: Normalized MDSC frequencies
