# Supplementary material for: Toward harmonized phenotyping of human myeloid-derived suppressor cells by flow cytometry: results from an interim study
Source: Cancer Immunol Immunother. 2016 Jan 4;65(2):161–9. doi: 10.1007/s00262-015-1782-5 (PMC4726716; doi:10.1007/s00262-015-1782-5)
Supplement: Supplementary file 1 — Supplementary material 1 (PDF 261 kb) [file 262_2015_1782_MOESM1_ESM.pdf]

		ANALYSIS WITHOUT DCM							ANALYSIS WITH DCM					
		MEAN	SD	%CV	Q1	MEDIAN	Q3		MEAN	SD	%CV	Q1	MEDIAN	Q3
DONOR 1 (HBC-480)	MDSC 1	2,66	4,86	182,62	0,08	0,31	2,24	MDSC 1	2,13	4,69	219,60	0,03	0,07	1,77
	MDSC 2	1,27	3,21	253,49	0,02	0,07	0,46	MDSC 2	2,17	6,76	311,68	0,01	0,03	0,11
	MDSC 3	0,53	0,43	81,90	0,09	0,54	0,91	MDSC 3	0,42	0,43	102,11	0,04	0,20	0,78
	MDSC 4	6,11	5,22	85,46	3,41	4,67	7,65	MDSC 4	5,43	4,06	74,81	3,22	4,52	7,34
	MDSC 5	1,04	2,80	268,88	0,12	0,24	0,47	MDSC 5	2,62	9,62	366,88	0,04	0,14	0,27
	MDSC 6	1,48	3,08	208,41	0,13	0,27	0,38	MDSC 6	3,97	16,44	414,37	0,08	0,14	0,26
	MDSC 7	4,67	3,98	85,22	1,98	3,60	5,68	MDSC 7	4,82	3,69	76,68	2,41	4,08	6,16
	MDSC 8	4,13	11,62	281,45	0,05	0,31	1,62	MDSC 8	4,15	13,70	330,41	0,04	0,12	0,59
	MDSC 9	2,35	2,39	101,58	0,83	1,23	3,52	MDSC 9	3,94	7,26	184,32	0,93	1,31	3,97
	MDSC 10	1,74	3,54	204,17	0,51	0,91	1,43	MDSC 10	3,24	8,28	255,84	0,38	0,92	1,57
DONOR 2 (HBC-514)	MDSC 1	4,12	8,27	200,75	0,08	0,44	3,95	MDSC 1	3,34	8,20	245,75	0,04	0,11	1,83
	MDSC 2	1,61	4,95	307,29	0,01	0,10	0,39	MDSC 2	1,37	5,13	375,14	0,00	0,02	0,12
	MDSC 3	0,81	0,92	113,94	0,06	0,37	1,33	MDSC 3	0,59	0,68	114,77	0,03	0,22	1,32
	MDSC 4	11,61	7,57	65,22	5,89	12,06	16,38	MDSC 4	10,72	6,88	64,13	5,65	9,81	14,53
	MDSC 5	0,77	2,28	296,86	0,04	0,12	0,23	MDSC 5	0,63	1,99	314,50	0,02	0,06	0,14
	MDSC 6	0,86	1,95	227,00	0,04	0,12	0,20	MDSC 6	0,37	0,91	246,77	0,02	0,04	0,12
	MDSC 7	9,35	7,13	76,28	3,57	9,17	11,91	MDSC 7	9,92	7,78	78,49	3,58	8,82	13,08
	MDSC 8	3,03	9,17	302,59	0,03	0,12	1,16	MDSC 8	1,08	2,64	245,05	0,02	0,04	0,14
	MDSC 9	2,98	3,07	103,00	0,82	1,76	4,44	MDSC 9	3,31	3,09	93,54	1,09	2,06	5,26
	MDSC 10	2,43	3,87	159,45	0,55	1,42	2,22	MDSC 10	1,85	3,17	170,91	0,32	1,03	1,63
DONOR 3 (L29_3)	MDSC 1	3,59	7,16	199,34	0,08	0,68	2,46	MDSC 1	2,80	6,70	238,90	0,04	0,10	2,28
	MDSC 2	1,41	3,80	269,61	0,04	0,12	0,34	MDSC 2	1,09	3,51	321,70	0,01	0,03	0,12
	MDSC 3	1,45	1,39	96,09	0,17	1,21	2,44	MDSC 3	1,21	1,35	111,98	0,06	0,36	2,22
	MDSC 4	11,50	6,63	57,65	5,72	11,95	17,92	MDSC 4	10,94	6,49	59,30	6,14	11,59	15,62
	MDSC 5	1,19	2,77	232,94	0,15	0,48	0,69	MDSC 5	0,52	1,02	195,91	0,11	0,19	0,43
	MDSC 6	1,78	3,62	202,99	0,20	0,41	1,08	MDSC 6	0,73	1,82	247,01	0,07	0,13	0,43
	MDSC 7	10,53	7,89	75,00	2,66	8,98	16,84	MDSC 7	10,55	7,81	73,99	2,78	10,12	15,87
	MDSC 8	3,25	7,15	219,80	0,15	0,37	2,59	MDSC 8	1,55	3,40	218,47	0,06	0,16	0,44
	MDSC 9	3,07	2,84	92,53	0,87	1,54	5,62	MDSC 9	3,27	2,76	84,59	0,91	1,50	6,14
	MDSC 10	3,05	4,70	154,07	0,60	1,85	3,36	MDSC 10	2,64	3,82	144,49	0,38	1,77	3,11

Supplementary Table 1

Descriptive statistics of the frequencies of ten MDSC subsets normalized on the count of lymphocytes + monocytes and identified either in presence or absence of DCM. For each donor and each myeloid subset, mean, standard deviation (SD), first and third quartile (Q1 and Q3, respectively) and median values are shown. Results produced in the same laboratory using either one 8-color panel or three 4-color panels were considered as independent values leading to 26 measurements for each subset. n=23 evaluable laboratories for analysis in the absence of dead cell marker, n=24 evaluable laboratories for analysis considering the dead cell marker. Evaluable laboratories were those that reported all requested data points.
